# Building Capacity for Prevention of Gender-Based Violence in the School Context

**DOI:** 10.3389/fpsyg.2021.720034

**Published:** 2021-10-11

**Authors:** Dean Ajduković, Ivana Car, Helena Päivinen, Anna Sala-Bubaré, Berta Vall, Marita Husso

**Affiliations:** ^1^Department of Psychology, Faculty of Humanities and Social Sciences, University of Zagreb, Zagreb, Croatia; ^2^The Unit of Social Research, Faculty of Social Sciences, Tampere University, Tampere, Finland; ^3^Department of Psychology, Sports and Educational Sciences, Blanquerna, Ramon Llull University, Barcelona, Spain

**Keywords:** gender-based violence, international comparison, needs-assessment, school context, training development

## Abstract

School-related gender-based violence (SRGBV) is highly prevalent worldwide which calls for a range of early prevention and innovative solutions. The presence of GBV in the school context is well-documented and it highlights the importance of building competencies of teachers and other school professionals for recognizing and intervening in SRGBV cases. This paper analyses the current and future teachers' training needs, and their level of preparedness for detecting and intervening in cases of GBV in the school context, with the objective of developing a targeted training program. The participants in this study were 597 current and future teachers and other school professionals from Croatia, Finland, and Spain. An *ad-hoc* built questionnaire was distributed in the three participating countries. Results show that the interest in receiving training is related to the perceived importance of coping with GBV in the (future) work and that the main topics of the training should focus on addressing parties of SRGBV, guidelines for prevention and intervention in schools as well as online GBV. These findings were similar in three countries, and they provided user-generated topics and tools that served as a guideline for the development of a training program that aims to increase the knowledge about SRGBV and to develop skills for coping with GBV in the school context regarding victims, bystanders and perpetrators.

## Introduction

School-related gender-based violence (SRGBV) occurs in or around schools and refers to sexual, physical, or psychological violence, perpetrated as a result of gender norms and stereotypes (UNESCO and UN Women, 2016). SRGBV often includes verbal aggression (emotional threats, insults, and humiliation), physical aggression (pushing, pulling, pinching, slapping) and sexual and economic violence (Ajduković, 2012). It can take place face-to-face, behind one's back, or online. Sometimes it may meet criminal characteristics (for example, assault and rape), but this is not always the case. Additionally, stereotypes that narrow the expression of gender and sexuality can be seen as part of the wider SRGBV spectrum as they can violate the pupils' right to integrity and self-determination, as well as the right to experience and define one's own gender.

These forms of violence are present in pupils' interactions and in adolescent romantic relationships. SRGBV is highly prevalent which was demonstrated in the large study conducted by the European Fundamental Rights Agency (FRA, 2014). It may start at the school-age and continue into adulthood. For example, in Finland, 47% of women report experiences of physical and/or sexual violence since the age of 15, 11% of women reported some form of sexual violence, 46% physical violence, and 10% psychological violence by an adult family member or a relative before the age of 15 (FRA, 2014). Another study in Croatia found that 86% of adolescents in a romantic relationship experienced some form of violence and that 93% of participants were perpetrators of some form of violence in a romantic relationship in the last 6 months (Ajduković et al., 2011). The recent Health Promotion study (Ikonen and Helakorpi, 2019) showed that every fourth pupil of the age of 14 had experienced sexual propositions or harassment during the past year where, most commonly, sexual harassment took place via mobile phone or internet (14–17%).

The importance of dealing with this topic is also demonstrated by the finding that GBV is linked to negative health behavior outcomes such as smoking and using drugs and alcohol (Smith et al., 2018) and chronic consequences such as post-traumatic stress disorder, gastrointestinal and cardiovascular problems (Basile and Smith, 2011) as well as avoiding school or completely dropping out (UNESCO and UN Women, 2016). Gruber and Fineran (2016) described that sexual harassment and bullying in general, both affect school attachment and academic performance but in different ways. In their study, sexual harassment had a broader range of impact than bullying in general, and emerged as the better predictor of school alienation, undermined relationships with teachers and other students and academic achievement. All forms of violence can be interconnected, and experiencing, perpetrating, or witnessing violence present a risk factor (Morrison et al., 2007) and can lead to more violence (Wilkins et al., 2014). Other risk factors associated with SRGBV can be categorized into the individual level, relationship level, community level and societal level of the ecological model, where learning about SRGBV phenomena as a protective factor operates at all these levels (Morrison et al., 2007). This highlights the importance of preventative educational interventions.

SRGBV can occur throughout the entire period of schooling but is mostly present during adolescence. An increase in sexual harassment is noticed between the ages of 12 and 16 (Gruber and Fineran, 2007). Ikonen and Helakorpi suggest that girls have more experience of sexualized violence and harassment than boys, as well as violence in the family, and boys had experienced more threat of physical violence (Ikonen and Helakorpi, 2019). It is not surprising that a number of preventions programs, such as the Fourth R, Safe Dates, Shifting Boundaries, WiseGuyz and Expect Respect, target this population (Crooks et al., 2019). These programs generally raise awareness of healthy and abusive behavior and focus on knowledge, attitudes and/or skills for coping with violence. Programs differ in their specific contents, but are generally found to be effective and resulting in increased awareness, reduced victimization and/or increased active bystander behavior (Crooks et al., 2019) which highlights the importance of creating and implementing such programs.

Furthermore, it has been demonstrated that the effectiveness of the SRGBV programs is also linked to their implementation and the person who is delivering the training (Cahill et al., 2019). In the school context, the program implementation is linked to teachers' characteristics, especially to their previous participation in other programs, and their knowledge and attitudes about the program's topic (Ollis, 2014). Implementation is easier for those who have experience with similar programs, sufficient knowledge about the topic and positive attitudes related to the topic. Another paper focused on SRGBV training implementation, suggests that teachers should use a positive communication style (taking interest in students, providing support, hope and encouragement, showing respect), and positive disciplines (strengthening positive behaviors and teaching about rights, responsibilities, rules and standards) in order to build the capacity of students to take responsibility for their own behavior (UNESCO, 2019) and to be perceived as an adult that pupils can come to and share their violent experience. Furthermore, it is important to highlight that the teachers are more likely to become personally involved in a violent incident if they do not view violent behavior as normative (Anagnostopoulos et al., 2009). These findings emphasize the importance of the school setting to prevent and mitigate SRGBV and that training teachers and school professionals is an investment with both short-term and as well as long-term benefits. In addition, teachers reported that engaging in educational interventions helped them develop trustworthy relationships with students and considered them important (Ollis, 2014). Engaging in such interventions is beneficial for the students since early sexual violence prevention interventions for high school and elementary school students could be effective in reducing and preventing sexual violence at higher education institutions (Steele et al., 2020).

This paper has two main objectives, first to explore the current and future teachers' training needs to tackle SRGBV. In this context, it also analyzes their level of preparedness for detecting and intervening in cases of GBV in the school context, their attitudes toward GBV and predictors of the interest in such training. The second objective is to determine whether the same training could be implemented in the three countries based on an analysis of differences and similarities in training needs between the countries. Findings should provide guidance for developing a training programme on SRGBV for current and future teachers and other school professionals.

## Method

### Participants

The convenience sample included a total of 597 participants who took part in the survey. Of them, 56.8% were school teachers and other school professionals, and 43.2% were students of these disciplines. The majority of the teachers and other school professionals (96%) reported the age range of 26–65 years which corresponds to the expected range for this profession. Also, the majority of students (96%) were in the 18–25 age category (80%) and 26–35 years (16%), which is expected for this group. The majority of students were female (86%) as well as the majority of teachers and other school professionals (85%). The gender proportion and age distributions correspond to the expected ones for these two populations which implies that the representativeness of the sample should be acceptable These were very similar in all three countries. A detailed description of the participants' characteristics is presented in Table 1.

**Table 1:** Sociodemographic characteristic of the participants (*N* = 597).

**Characteristics**		***f***	**%**
Age	18–25	180	30.2
	26–35	149	25.0
	36–45	112	18.8
	46–55	100	16.8
	56–65	53	8.9
	Over 65	3	0.5
Gender	Female	486	81.4
	Male	99	16.6
	Other	3	0.5
	Refuse to answer	9	1.5
Role	Student Teacher or school professional	258 339	43.2 56.8

### Instruments

The questionnaire we used was created for the purpose of this study. It was jointly developed by the research team from all three countries. The content of the questionnaire was based on 13 focus group discussions with representatives of the target groups and literature scoping. Qualitative thematic analysis of the focus groups transcripts provided the specific contents for formulating the survey questions. This analysis showed a high level of consistency among the three countries. Furthermore, we used the standard back-translation method in the respective languages to ensure the full equivalency of the items. The questionnaire examined topics of coping with GBV in the school context, strategies for intervening in cases of SRGBV, and sociodemographic characteristics of participants. Each of the topics was tapped by a few multiple-choice or Likert-scale questions.

*Coping with SRGBV* includes two scales. *Support in coping with SRGBV* is a Likert type scale. It contains nine items, such as *There is enough time to carry out tasks related to cases of SRGBV*. The response format was 1–5 (from “Completely disagree” to “Completely agree”). The higher score indicates having a higher level of support in coping with SRGBV. The total score is a sum of responses on nine items. The overall mean of the scale was 24.1 (*SD* = 7.56). Cronbach's alpha for the scale was 0.90 which implies a high internal consistency. The scale is unifactorial and the first factor explains 57.4% of the variance. The other scale is *Coping with SRGBV Scale*, also comprising 9 items and the same response format. An example of an item is: *I know where to find guidelines on addressing SRGBV when I need them*. The higher score indicates a higher level of readiness for coping with SRGBV. The overall mean of the scale was 24.9 (*SD* = 7.62), with Cronbach's alpha of 0.90 which shows a high internal consistency of the scale. The scale is unifactorial and the first factor explains 57.6% of the variance.

*Strategies for intervening with cases of SRGBV* included items about tools and protocols for screening for SRGBV, experience in directing victims and perpetrators of SRGBV incidents to services, identification of SRGBV among or against students within the last 12 months, being prepared to identify and address SRGBV, and assessing the importance of SRGBV in current or future work. Among them, the two items “How important a part of your work is SRGBV?” and “How prepared do you feel to identify and address SRGBV in your students?” were presented with a response format 1–5 (from not at all important/prepared to very important/prepared). The three questions “Have you identified SRGBV among or against students within the last 12 months?,” “What kind of experience do you have in directing victims and perpetrators of SRGBV incidents to services?” and “Do you use tools or protocols developed for asking and screening for SRGBV?” were multiple choice answers. These answers can be seen in **Table 4**.

*Sociodemographic characteristics* included questions on age, gender and current job position with multiple-choice responses which can be seen in Table 1.

### Procedure

Data was collected through an online survey. The link to the questionnaire was shared via online social networks that gather specific target groups of interest (for example, the Creative teachers' group). In addition, the invitation to participate was sent via e-mail addresses for greater data heterogeneity. The questionnaire included the invitation to participate in the survey with an explanation of the purpose, data protection protocol, and a thank you note with an e-mail address that the participants could contact if they had any comments or questions. Participants were informed about the goals of the study, the ability to stop their participation at any time, and their right to retract their data. All participants gave their informed consent before starting the survey. The study was reviewed and approved by the respective institutional ethic boards.

Data collection lasted during the spring and fall of 2020 in the three participating countries.

## Results

### Needs-Assessment

The first objective of this study was to analyze the needs for training on GBV in current and future teachers and other school professionals. The assessment of training needs was conducted separately for the two groups of participants.

Results show that students, future teachers, and other school professionals, estimate that they are underprepared to deal with cases of GBV (*M* = 2.9, *SD* = 1.02), and believe that dealing with such cases will be a highly important part of their future work (*M* = 4.2, *SD* = 1.18). They state that they are interested in attending training on SRGBV (*M* = 4.3, *SD* = 0.94). In addition, they are most interested in learning about identifying and addressing parties of SRGBV, guidelines for prevention and intervention in schools, and gender-based violence that takes place online.

Teachers and school professionals estimate that they are moderately prepared to deal with cases of GBV (*M* = 3.1*, SD* = 1.02), and believe that dealing with such cases is a relatively important part of their work (*M* = 3.9, *SD* = 1.11). They are interested in attending training on this topic (*M* = 4.0, *SD* = 1.12). Furthermore, they are most interested in learning about guidelines for prevention and intervention in schools, identifying cases of SRGBV, and gender-based violence that takes place online.

Additionally, 55% of teachers and school professionals reported that they are not aware of the tools and protocols intended to identify GBV, 33% know about them but do not use them, and only 11% use such tools. Within the last 12 months of their work, 33% of teachers and school professionals identified victimization in school, 23% identified perpetrations, and 25% identified witnessing of SRGBV incidents. However, 54% of teachers and school professionals reported that they did not identify a case of SRGBV. Also, when asked about referring cases to services, 40.4% of teachers and school professionals did not know how to refer students or their parents or guardians to child protection or other services if there was a need to do it, 8.9% claimed that such services do not exist, and 6.5% did not think that it is their responsibility. In this group, 19.8% stated that it has not been necessary to direct students (or their parents/tutors) to any services and 24.4% had experience in referring to child protection or other services.

### Predicting Interest for Training on SRGBV

In order to examine what are the predictors of interest for the training on SRGBV, two hierarchical regression analyses, for the group of teachers and for the group of students, were conducted.

Results of the hierarchical regression analysis for the group of students are shown in Table 2. In the first step of the regression analysis in the group of students, the importance of SRGBV was introduced as a predictor. This predictor had a significant value in explaining the variance of the interest for the training (*β* = 0.464, *p* < 0.01). Those who perceived the topic of SRGBV as more important also had a higher interest in the training. In that step, the 21% variance of the criterion was explained. In the second step, the preparedness for coping with SRGBV was entered. Contrary to our expectations, this predictor was not significant for explaining the variance in the interest in training on SRGBV (*β* = −0.042, *p* > 0.05).

**Table 2:** Prediction of interest for training on GBV for students.

**Criterion: Interest in training on GBV**		
	**1. step**	**2. step**
	***β***	***β***
Importance of GBV	0.464^**^	0.470^**^
Preparedness for coping with GBV		−0.042
*R^2^*	0.212	0.211
*F*	68.28^**^	34.36^**^
Δ *R*^2^		0.002
*F* Δ *R*^2^		0.557

** *p < 0.01*.

Results of the hierarchical regression analysis for the group of teachers and other school professionals are shown in Table 3. In the analysis for the group of teachers and other school professionals, predictors of interest for the training on SRGBV, in the first step were the importance of SRGBV and preparedness for coping with SRGBV. Contrary to our expectations, preparedness for coping with SRGBV was not a significant predictor of interest for the training (*β* = −0.023, *p* > 0.05). In contrast, the importance of SRGBV was a significant predictor (β = 0.377, *p* < 0.01). Those who perceived the topic of SRGBV as more important also had a higher interest in further training. In that step, the 13% variance of the criterion was explained. In the second step, the Coping with SRGBV and Support in coping with SRGBV were introduced as predictors. These variables were not significant predictors of interest for the training on SRGBV (*β* = 0.068; *p* > 0.05, *β* = −0.014, *p* > 0.05). This regression model explained 13% of the variance of interest for the training on SRGBV.

**Table 3:** Prediction of interest for training on GBV for teachers and other school professionals.

**Criterion: Interest in training on GBV**		
	**1. step**	**2. step**
	***β***	***β***
Importance of GBV	0.377^**^	0.374^**^
Preparedness for coping with GBV	−0.023	−0.052
Coping with GBV		−0.014
Support in addressing GBV		0.068
*R^2^*	0.132	0.129
*F*	24.94^**^	12.69^**^
Δ *R*^2^		0.003
*F* Δ *R*^2^		0.511

** *p < 0.01*.

### Cross-Country Comparison

The second objective of this study includes an international comparison of the training needs. In order to answer the question would the same training be appropriate in all three countries, differences and similarities in the training needs between the three countries were analyzed. Croatian, Finnish, and Spanish results were compared separately for the groups of current and future teachers and school professionals. The variables of preparedness for coping with SRGBV and the importance of SRGBV were analyzed by ANOVA test.

The results showed that there is no difference in preparedness for coping with SRGBV among students in the three countries (*F* = 0.56, *p* > 0.05). However, there were significant differences in the perceived importance of SRGBV (*F* = 13.68, *p* < 0.01). *Post-hoc* Scheffe test determined that the perceived importance of SRGBV is higher among Finnish students than Croatian and Spanish students. Furthermore, the teachers' perception of the importance of SRGBV in the three countries did not differ (*F* = 0.61, *p* > 0.05). They see SRGBV as an equally important topic in all three countries. However, analysis of preparedness for coping with SRGBV has shown differences (*F* = 12.48, *p* < 0.01) and the *post-hoc* test revealed differences between Finnish teachers' preparedness compared to their Croatian and Spanish counterparts. Finnish teachers are feeling more prepared for coping with SRGBV than Croatian and Spanish teachers.

Furthermore, the international comparison included part of the descriptive data. Dominant answers on the multiple-choice questions on tools and protocols, directing students to services and identification of SRGBV cases were analyzed by examining the dominant answer to those questions in all countries. Regarding the use of tools and protocols for the identification of SRGBV cases, teachers in all three countries most often state that they do not know about them. Also, teachers in all three countries had experience in directing students to services or stated that it was not necessary to direct students to services as a second dominant answer. Another similarity was that the teachers and other school professionals in all three countries did not identify cases of SRGBV in the last 12 months. More detailed results are presented in Table 4.

**Table 4:** Strategies for intervening with cases of SRGBV.

**Subsample**		**Croatia**	**Spain**	**Finland**
		**%**	**%**	**%**
Identified GBV among students within the last 12 months	I have not identified GBV Victimization Perpetration Witnessing of GBV incidents	46.2 20.2 13.9 19.7	25.7 22.9 13.3 13.3	47.6 40.2 32.9 20.7
Use of tools or protocols for screening for GBV	I do not know about them I know about them, but I do not use them I use such tools or protocols	50.6 39.7 9.7	28.6 17.1 11.4	69.5 24.4 6.1
Experience in directing victims and perpetrators of GBV (or their parents/tutors) incidents to services	Directed to child protection or other specialized services It has not been necessary to direct to any services I don't know how to direct them There are no services available It is not my responsibility I don't want to answer	33.9 34.9 16.7 5.9 9.1 9.4	28.6 17.1 12.4 1.9 6.7 1.0	47.3 39.0 19.5 3.7 3.7 2.4

## Discussion

This study is focused on the needs for further training and what specific contents of such training on SRGBV would be interesting and useful for teachers and future teachers and other school professionals. Results showed that the current and future teachers do not feel prepared to deal with cases of SRGBV. This is in accordance with other findings that suggest that teachers and other school staff are underprepared to deal with SRGBV (Anagnostopoulos et al., 2009) and that education on this topic should be also included in the teachers' studies before first work experience (UNESCO and UN Women, 2016). On the positive side, teachers and the future teachers are very interested to undertake training on SRGBV. This further highlights the need for creating such training and incorporating them in the school curricula, since they are one of the key components of addressing GBV in educational settings (Sida, 2015). Results suggest that they would be most interested in learning about identifying and addressing parties of SRGBV, guidelines for prevention and intervention in schools, and GBV that takes place online. Training should also include information about tools and protocols intended to identify GBV since a high percentage of school teachers and other school professionals did not know about them. Furthermore, 54% of teachers and school professionals did not identify SRGBV in the last 12 months of their work. Thus, in further training, it would be important to include the information on types and forms of SRGBV, to ease the identification of cases which is especially important since it is under-reported (Tsouroufli, 2020). The training programme should also include information on directing students and their guardians to specialized services if there is a need for this. This is especially important since 40% of teachers and other school professionals are not experienced in directing to specialized services.

Furthermore, an important predictor of the interest for the training on SRGBV is the perceived importance of SRGBV in (future) work. This implies that raising awareness of SRGBV, both within the professional training and public campaigns on television and social media could be useful (Morrison et al., 2007), and is in accordance with the guideline that raising awareness is an important part of the training (Sida, 2015). On the other hand, the total variance explained of the interest for the training is relatively small. This indicates that other variables, such as personal characteristics or situational factors (personal experience with GBV, school support for training attendance), could be included in further research.

Based on these findings we concluded that the training programme should cover the topics of knowledge about SRGBV, awareness of and reflection on own relation to SRGBV, skills for prevention and recognition of the cases of SRGBV, online GBV, and legal framework for dealing with such cases. The programme should focus on skills building, deeper knowledge and changing attitudes as three main categories of learning outcomes. It is expected that gain in these three components should also encourage the current and future teachers and other school professionals to intervene and become personally engaged in addressing cases of SRGBV in their workplace.

Furthermore, we found that the same training would be appropriate for use in the three countries. There were no differences in preparedness for coping with SRGBV among students in different countries and they felt equally prepared. However, the importance of SRGBV was higher among Finnish students than Croatian and Spanish students. Likewise, Finnish teachers felt slightly more prepared for coping with SRGBV than Croatian and Spanish teachers. Nevertheless, the perceived importance of SRGBV in the teachers' group was the same in the three countries, and they saw SRGBV as an equally important topic. Additionally, the dominant answers regarding the tools and protocols, identification of the SRGBV cases and directing to services were the same in all three countries. Teachers and future teachers in all three countries dominantly reported that they do not know about tools and protocols for the identification of SRGBV cases, but that they have some experience in directing students to other services, and that they most often did not identify cases of SRGBV during the past year. On the whole, despite minor differences, these findings indicate that the same training programme would be suitable for all three countries. The international training programme would be in accordance with a guideline that international collaboration across Europe is important to improve the longer-term well-being of parties of GBV (Bradbury-Jones et al., 2017).

### Limitations and Future Directions

Although this study presents evidence-based findings for the development of an international training programme, it has some limitations. It is important to acknowledge that the online questionnaire was posted via online social networks. Participants who are members of such networks may differ from those who do not use such tools, as Facebook, which limit the possibility to generalize these findings. In addition, there is also a risk of self-selection of participants, which is always the case in online surveys. It is possible that participation was accepted by those who are more interested in the topic and potentially differ in other relevant characteristics. Thus, it is possible that those who find this topic repulsive or controversial did not engage in completing the questionnaire. This could have distorted the results and limited the possibility of generalizing these findings. Also, recollection was necessary to answer the questionnaire which may be distorted in recalling and quantifying SRGBV cases. Recalling the average school day could be further complicated by the “work from home” situation during the Covid-19 pandemic. Namely, the collection of data for this research took place while the measures on restricting movement and work due to the coronavirus pandemic were in force and the educational institutions were closed. Students attended classes online, and interaction with teachers was maintained through video calls. Online teaching further limits identification and dealing with SRGBV cases. Finally, a relatively small proportion of male teachers participated in the research, but this reflects the gender bias in the profession.

In future studies, it would be desirable to ensure greater representativeness of the sample and include some individual and contextual variables.

Nevertheless, these findings indicate the needs of future and current teachers and other school professionals for improved support in coping with GBV in the school context and provide the basis for creating international training on SRGBV that could serve this purpose.

## Data Availability Statement

The raw data supporting the conclusions of this article will be made available by the authors, without undue reservation.

## Ethics Statement

The studies involving human participants were reviewed and approved by Ethics Committee, Department of Psychology, Faculty of Humanities and Social Sciences, University of Zagreb. The patients/participants provided their written informed consent to participate in this study.

## Author Contributions

IC, AS-B, and HP performed data analysis. IC and DA drafted the manuscript. The manuscript was commented on and approved by all authors. All authors worked on the study design and data collection.

## Funding

This research is funded by the Rights, Equality and Citizenship Programme of the European Union (2014–2020) under grant agreement no. 856816.

## Conflict of Interest

The authors declare that the research was conducted in the absence of any commercial or financial relationships that could be construed as a potential conflict of interest.

## Publisher's Note

All claims expressed in this article are solely those of the authors and do not necessarily represent those of their affiliated organizations, or those of the publisher, the editors and the reviewers. Any product that may be evaluated in this article, or claim that may be made by its manufacturer, is not guaranteed or endorsed by the publisher.
